# Serum Carcinoembryonic Antigen Levels Across Molecular Subtypes and Their Clinical and Prognostic Implications in Metastatic Non-Small Cell Lung Cancer

**DOI:** 10.3390/medicina62040718

**Published:** 2026-04-09

**Authors:** Ali Aytac, Bilgin Demir, Meltem Demirtas Gulmez, Hayati Arvas, Tuba Ugur Tuzcu, Enes Erul, Salih Tunbekici, Tahir Yerlikaya, Sezai Tunc, Halil Ibrahim Ellez, Yasemin Aydinalp Camadan, Kubra Canaslan, Rumeysa Colak, Zuhat Urakci, Elif Berna Koksoy, Ozan Yazici, Ali Alkan, Ozgur Tanriverdi, Erdem Goker, Ahmet Demirkazik

**Affiliations:** 1 Medical Oncology Clinic, Mehmet Akif Inan Training and Research Hospital, University of Health Sciences, Sanliurfa 63320, Turkey; 2 Division of Medical Oncology, Department of Internal Medicine, Faculty of Medicine, Aydın Adnan Menderes University, Aydın 09100, Turkey; 3 Division of Medical Oncology, Department of Internal Medicine, Faculty of Medicine, Dicle University, Diyarbakır 21000, Turkey; 4 Division of Medical Oncology, Department of Internal Medicine, Faculty of Medicine, Gazi University, Ankara 06000, Turkey; 5 Division of Medical Oncology, Department of Internal Medicine, Faculty of Medicine, Ankara University, Ankara 06000, Turkey; 6 Division of Medical Oncology, Department of Internal Medicine, Faculty of Medicine, Ege University, İzmir 35000, Turkey; 7 Medical Oncology Clinic, Antalya State Hospital, Antalya 07000, Turkey; 8 Division of Medical Oncology, Department of Internal Medicine, Faculty of Medicine, Harran University, Şanlıurfa 63320, Turkey; 9 Division of Medical Oncology, Department of Internal Medicine, Faculty of Medicine, Muğla Sıtkı Koçman University, Muğla 48000, Turkey

**Keywords:** carcinoembryonic antigen (CEA), metastatic non-small cell lung cancer, molecular subtypes, oncogenic driver mutations, prognosis, overall survival

## Abstract

*Background and Objectives*: Serum carcinoembryonic antigen (CEA) is a widely used biomarker in non-small cell lung cancer (NSCLC). However, its association with oncogenic driver alterations and prognostic significance across molecular subtypes in metastatic disease remains insufficiently defined. *Materials and Methods*: This retrospective multicenter study included 332 patients with metastatic NSCLC harboring oncogenic alterations (EGFR, ALK, ROS1, KRAS, and others) from eight oncology centers in Türkiye. Baseline serum CEA levels measured at metastatic diagnosis were analyzed on the natural logarithmic scale. Associations between CEA levels, molecular subtypes, clinical features, and overall survival (OS) were evaluated using generalized linear models and Cox proportional hazards regression. *Results*: Baseline CEA levels differed significantly across molecular subtypes (*p* = 0.001), with EGFR-mutant tumors showing the highest median levels. Multivariable analysis identified driver alteration, histology, and metastatic burden as independent determinants of baseline CEA. Higher baseline CEA and metastatic site count were independently associated with increased mortality risk (HR 1.151 and 1.279 per unit increase, respectively; *p* < 0.001), while female sex was protective (HR 0.626; *p* = 0.004). KRAS mutations were associated with poorer survival compared with EGFR (HR 2.370; *p* < 0.001). Kaplan–Meier analyses showed a consistent trend toward longer OS in patients with CEA < 5 ng/mL, with significance only in the rare alteration subgroup. *Conclusions*: Baseline CEA may reflect underlying tumor biology across molecular subtypes and are associated with survival outcomes in metastatic NSCLC. However, given the variability across subgroups and modest effect sizes, these findings should be interpreted with caution. Prospective studies evaluating longitudinal CEA dynamics are warranted.

## 1. Introduction

Lung cancer remains the leading cause of cancer-related mortality worldwide, accounting for approximately 1.8 million deaths each year. Non-small cell lung cancer (NSCLC) accounts for nearly 85% of all lung cancer cases and is frequently diagnosed at an advanced or metastatic stage, where systemic therapy is the mainstay of treatment [1] Over the past two decades, advances in molecular oncology have dramatically transformed the therapeutic landscape of metastatic NSCLC. The identification of oncogenic driver alterations, including mutations or rearrangements in epidermal growth factor receptor (EGFR), anaplastic lymphoma kinase (ALK), proto-oncogene tyrosine-protein kinase ROS (ROS1), kirsten rat sarcoma virus oncogene homologue (KRAS), B-Raf proto-oncogene (BRAF), mesenchymal epithelial transition factor receptor (MET), and other oncogenic alterations, has enabled the development of targeted therapies that significantly improve progression-free survival, overall survival (OS), and quality of life compared to conventional chemotherapy [2,3].

Comprehensive molecular profiling using next-generation sequencing (NGS) is currently the standard diagnostic approach for detecting driver mutations in advanced NSCLC [4]. However, several challenges continue to limit its universal implementation, including insufficient tumor tissue, procedural risks related to invasive biopsies, extended turnaround times, and financial or logistical constraints, particularly in resource-limited healthcare settings. Consequently, the identification of accessible, noninvasive, and cost-effective biomarkers that may provide early clues regarding tumor molecular characteristics remains an area of significant clinical interest.

Carcinoembryonic antigen (CEA) is one of the most widely used serum tumor markers in lung cancer, especially in adenocarcinoma. Elevated serum CEA levels are associated with advanced disease stage, higher tumor burden, metastatic dissemination, and inferior survival outcomes in patients with NSCLC [5,6]. In addition to its established prognostic role, emerging evidence suggests that CEA levels are associated with specific molecular subtypes of lung cancer. Several studies have reported significantly higher baseline CEA levels in patients harboring EGFR mutations than in those with wild-type tumors, suggesting a potential link between tumor biology and serum biomarker expression [7,8].

Despite these observations, the association between baseline serum CEA levels and the broader spectrum of molecular subgroups in metastatic NSCLC remains insufficiently characterized. Prior studies have largely emphasized the prognostic or treatment-monitoring utility of CEA, while comparative analyses across multiple driver-defined subgroups are scarce. Clarifying whether baseline CEA differs by driver subtype could provide additional insight into tumor biology and offer an easily accessible adjunct during the initial diagnostic work-up, particularly when tissue is limited or molecular results are pending. Therefore, we investigated baseline CEA levels across distinct oncogenic mutation subgroups in metastatic NSCLC, hypothesizing that CEA distributions vary by mutation subtype and may help refine clinical suspicion of the underlying molecular profile.

## 2. Materials and Methods

This retrospective, multicenter study included patients diagnosed with metastatic NSCLC in January 2010 and December 2025. Eight oncology centers across Türkiye participated in this study. Patients were identified using the institutional electronic medical record systems at each participating center. Individuals with available baseline CEA measurements at the time of metastatic disease diagnosis were eligible for inclusion. Patients with incomplete clinical records or missing baseline CEA data were excluded. The study population consisted of patients with available molecular profiling and identified oncogenic alterations; therefore, patients without detectable driver alterations were not included, resulting in a molecularly selected cohort.

Demographic and clinical characteristics, including age, sex, smoking history, histopathological subtype and stage at diagnosis, were collected. Molecular alteration status was recorded for EGFR, ALK, ROS1, KRAS, and other less common oncogenic drivers, when available. The “other alterations” group was defined as a heterogeneous category including genomic alterations without established targeted therapies in metastatic NSCLC, such as TP53, PTEN, and PIK3CA.

Molecular testing was performed at the participating centers using validated methods, including polymerase chain reaction (PCR), fluorescence in situ hybridization (FISH), immunohistochemistry (IHC), and NGS, according to institutional standards and availability during the study period. PD-L1 expression was evaluated by IHC and reported as tumor proportion score (TPS). PD-L1 status was not available for all patients due to the retrospective design of the study, evolving clinical practice over time, variability in testing across centers, and limited access to PD-L1 testing in certain settings.

Treatment-related data including systemic therapy regimens, targeted therapies, immunotherapy use, and treatment sequencing were documented. Treatment decisions were made according to contemporary national and international guidelines and at the discretion of the physician.

Baseline laboratory parameters were retrieved from medical records, with particular emphasis on serum CEA levels measured at the time of metastatic diagnosis, prior to the initiation of systemic therapy. Laboratory measurements were performed at each participating center according to the local standard procedures. To further enhance comparability, laboratory reports were systematically reviewed and CEA values were standardized with respect to measurement units and reference ranges prior to analysis. No central laboratory calibration or assay harmonization was performed, and all measurements were based on local assays used at each participating center. To improve the data consistency across centers, all laboratory values were reviewed and harmonized before the analysis. When multiple baseline CEA measurements were available, the value closest to the date of metastatic diagnosis was selected.

A serum CEA cutoff value of 5 ng/mL was used, as it represents the widely accepted upper limit of normal in clinical practice and has been commonly adopted in previous studies evaluating its prognostic significance in non-small cell lung cancer [9,10,11].

Follow-up data, including treatment response, disease progression, and survival status, were obtained from institutional databases and patient follow-up records. Patients were followed up from the time of metastatic diagnosis until death or the last clinical contact. The median follow-up duration was calculated using the reverse Kaplan–Meier method. OS was defined as the time from the diagnosis of metastatic disease to death from any cause or the last clinical follow-up. Patients without documented deaths were censored on the date of the last contact.

To ensure data quality, anonymized datasets from the participating centers were reviewed by the coordinating center, and cross-validation procedures were performed to minimize data entry errors. Missing data were assessed for their frequency and patterns. Variables with substantial missingness (e.g., PD-L1) were excluded from multivariable analyses, and analyses were performed using an available-case approach for the remaining variables.

Potential sources of selection and information bias inherent to retrospective studies were minimized by predefined inclusion criteria and standardized data collection procedures across the centers. The study design, conduct, and reporting adhered to the Strengthening the Reporting of Observational Studies in Epidemiology (STROBE) guidelines.

### 2.1. Ethics Approval

This study was conducted in accordance with the Declaration of Helsinki and complied with national regulations. Ethical approval was obtained from the Clinical Research Ethics Committee of Harran University (approval number: HRÜ/25.19.58, date: 1 December 2025). Permissions to conduct the study were obtained from the chief physician’s office of each participating center. Owing to the retrospective nature of the study, the requirement for informed consent was waived. All patient data were anonymized before analysis.

### 2.2. Statistical Analyses

All analyses were performed using IBM SPSS Statistics v29 and R v4.5. Serum CEA exhibited a markedly right-skewed distribution; therefore, CEA was analyzed on the natural logarithmic scale (lnCEA) when treated as a continuous variable. For the Kaplan–Meier analyses, CEA was additionally categorized (CEA group < 5 ng/mL vs. ≥5 ng/mL) for clinical interpretability. The determinants of baseline CEA were assessed using a generalized linear model (GLM) with lnCEA as the dependent variable. Exponentiated coefficients are presented as geometric mean ratios (exp[B]) with 95% confidence intervals (CIs). To mirror a split-file approach, curves and log-rank (Mantel–Cox) tests were generated separately within each driver mutation subgroup. Prognostic factors for OS were evaluated using the Cox proportional hazards regression. Covariates were prespecified based on clinical relevance and supported by univariable screening, with model complexity constrained by the number of events and proportional hazards assumptions. The proportional hazards assumption was evaluated, and no violations were detected. Results are reported as hazard ratios (HRs) with 95% confidence intervals (CIs), and a two-sided *p*-value < 0.05 was considered statistically significant.

## 3. Results

### 3.1. Patients Characteristics

A total of 332 patients were included and grouped by driver alteration as follows: EGFR (*n* = 180), ALK (*n* = 45), ROS1 (*n* = 27), KRAS (*n* = 65), and other alterations (*n* = 15). The baseline characteristics of the driver subgroups are summarized in Table 1. Sex distribution differed across subgroups (*p* < 0.001), with a higher proportion of females in the EGFR-mutant group and a predominance of males in the KRAS-mutant group. The median age at diagnosis was approximately 62 years and varied by genotype (*p* < 0.001), with the youngest median age observed in ALK-positive patients. Smoking status also differed significantly (*p* < 0.001), with the lowest prevalence in EGFR-mutant and the highest in patients with KRAS mutations. Most tumors were adenocarcinomas, and histology varied across driver subgroups (*p* < 0.001). The metastatic patterns were broadly similar, although distant lymph node metastasis, pleural metastasis, and local lung recurrence differed according to the genotype.

**Figure 1:**
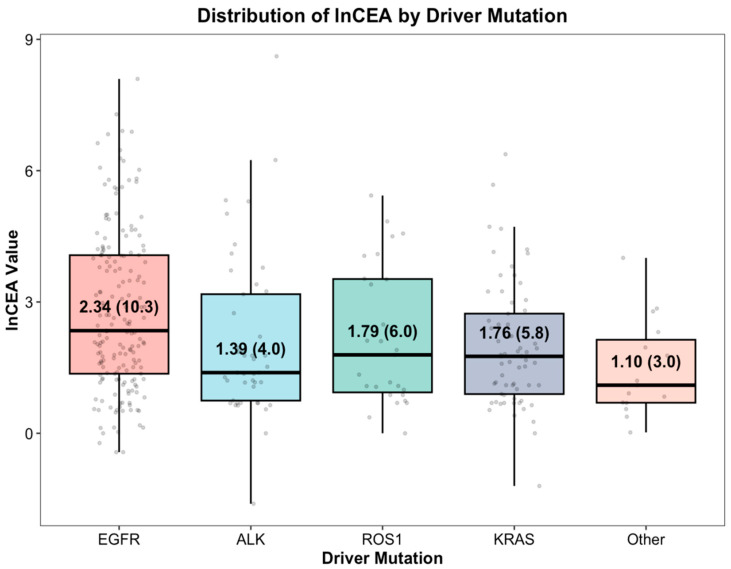
Distribution of ln(CEA) according to oncogenic driver mutation group.

The PD-L1 categories did not differ significantly (*p* = 0.317), with the PD-L1 status unknown in approximately half of the cases. First-line treatment varied by driver alteration (*p* < 0.001), whereas the median number of metastatic treatment lines was similar across the groups (median, 2; *p* = 0.368). Baseline CEA differed by driver subgroup (*p* = 0.001) and is visualized on the log scale in Figure 1.

Boxplots show the median (horizontal line), interquartile range (box), and 1.5 × IQR whiskers; points represent the individual patients. The labels indicate the group-specific median ln(CEA) with the corresponding median CEA. Because CEA values were highly right-skewed with extreme outliers, CEA was log-transformed for visualization and modeling; in regression analyses, lnCEA was used, and effect estimates were reported as geometric mean ratios (exp[B]).

### 3.2. Determinants of Baseline CEA

CEA values were highly right-skewed with extreme outliers; therefore, CEA was log-transformed for visualization and modeling (Figure 1). In the multivariable GLM for lnCEA (Table 2), driver alterations, histology, and metastatic site count were independently associated with lnCEA. Using EGFR as the reference, other/rare alterations were associated with lower CEA (GMR 0.36, 95% CI 0.14–0.92; *p* = 0.033), and ALK (GMR 0.50, 95% CI 0.28–0.89; *p* = 0.018) and ROS1 (GMR 0.55, 95% CI 0.32–0.97; *p* = 0.039) also showed lower CEA. Squamous histology was associated with lower CEA levels than adenocarcinoma (GMR 0.43, 95% CI 0.22–0.83; *p* = 0.012), and an increasing metastatic burden was independently associated with higher CEA levels (per additional metastatic site: GMR 1.21, 95% CI 1.03–1.41; *p* = 0.020). Smoking status was not significantly associated with lnCEA levels (*p* = 0.488).

### 3.3. Overall Survival and Prognostic Factors

**Figure 2:**
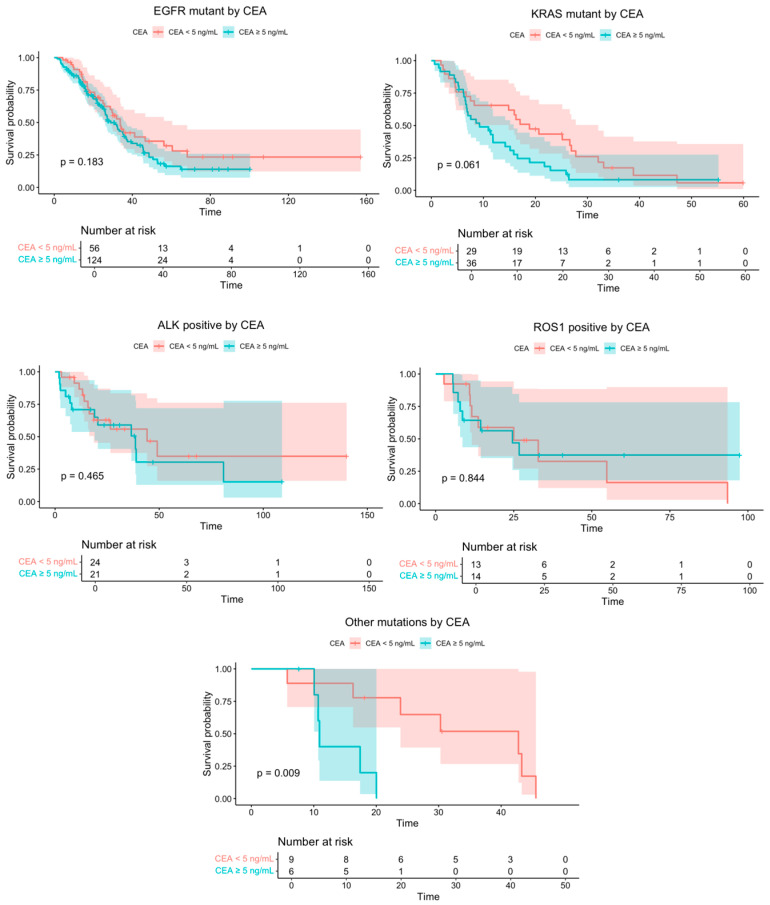
Kaplan–Meier overall survival curves according to baseline CEA levels (<5 vs. ≥5 ng/mL) across molecularly defined subgroups of metastatic NSCLC.

The median follow-up was 46.4 months (95% CI 34.9–57.8), during which 215 deaths occurred. The median OS for the overall cohort was 26.2 months (95% CI: 23.2–29.3). Across driver-defined subgroups, Kaplan–Meier estimates generally indicated longer OS for CEA < 5 ng/mL than for CEA ≥ 5 ng/mL, with a statistically significant difference observed only in the other alterations group. The median OS (CEA < 5 vs. ≥5) was 33.8 vs. 28.9 months in EGFR (*p* = 0.183), 44.2 vs. 38.6 months in ALK (*p* = 0.465), 25.0 vs. 24.6 months in ROS (*p* = 0.844), 18.9 vs. 9.3 months in KRAS (*p* = 0.061), and 42.8 vs. 10.9 months in the other group (*p* = 0.009) (Figure 2).

In the univariate Cox analyses (Table 3), higher lnCEA, higher metastatic site count, older age, male sex, smoking, and bone metastasis were associated with mortality, whereas first-line tyrosine kinase inhibitors (TKI) use was associated with lower mortality. Among the driver subgroups, KRAS was associated with higher mortality than EGFR (HR 2.69, *p* < 0.001), whereas ALK, ROS1, and other alterations did not differ significantly from EGFR.

In the multivariable Cox model (Table 4), higher lnCEA (HR 1.149 per 1-unit increase; *p* < 0.001) and greater metastatic site count (HR 1.273 per additional site; *p* < 0.001) were independently associated with higher mortality, while female sex was protective (HR 0.623; *p* = 0.004). Age, histology, and smoking were not significant. Using EGFR as the reference, KRAS was associated with higher mortality (HR 2.301; *p* < 0.001), whereas ALK, ROS1, and other alterations were not significantly different.

## 4. Discussion

In this multicenter cohort of patients with metastatic NSCLC harboring oncogenic driver alterations, we demonstrated that baseline serum CEA levels differ across molecular subtypes and were independently associated with OS. These findings indicate that CEA may reflect both tumor burden and underlying tumor biology, suggesting a broader clinical role beyond its traditional use as a tumor marker.

CEA has long been investigated as a prognostic biomarker for lung cancer. Several studies and meta-analyses have demonstrated that elevated pretreatment CEA levels are associated with worse survival outcomes in patients with NSCLC. In a large cumulative meta-analysis including 4296 patients, elevated baseline CEA was consistently associated with poorer overall survival, supporting its role as a prognostic biomarker in NSCLC [6]. Similarly, earlier meta-analytic evidence has shown that high serum CEA levels significantly increase the mortality risk in NSCLC, reinforcing the prognostic relevance of this marker in clinical practice [12]. Consistent with this literature, lnCEA remained independently associated with mortality after adjusting for driver mutations and metastatic burden.

A central finding of our study was the significant variation in baseline CEA levels among the molecularly defined subgroups. EGFR-mutant tumors exhibited higher baseline CEA levels than other driver alterations, aligning with prior evidence suggesting an association between elevated CEA and EGFR mutation status. Earlier studies reported that increasing serum CEA concentrations correlated with higher EGFR mutation rates, proposing that CEA may reflect specific biological characteristics of EGFR-driven adenocarcinoma [7,8,13]. Recently, a large meta-analysis including over 4000 patients demonstrated that CEA-positive NSCLC cases were more likely to harbor EGFR mutations, supporting the hypothesis that serum CEA may serve as an adjunctive clue to underlying molecular alterations [14]. In contrast to our findings, Uysal et al. [15] reported no significant association between baseline CEA levels and EGFR mutation status in a cohort of patients with advanced lung adenocarcinoma. This discrepancy may be explained by differences in study design and analytical approach. The study by Uysal et al. included a smaller cohort and primarily evaluated binary comparisons using predefined CEA cut-off values. In contrast, our multicenter study evaluated CEA as a continuous variable across multiple molecular subtypes within a multivariable framework. These methodological differences may account for the variation in findings. Our multicenter analysis extends these observations by demonstrating differences across multiple molecular subgroups rather than limiting the comparison to EGFR versus wild-type disease.

Beyond molecular subtypes, metastatic disease burden was independently associated with higher CEA levels, confirming previous reports linking serum tumor markers with the extent of systemic disease. The positive association between the number of metastatic sites and CEA levels supports the interpretation that elevated CEA levels reflect tumor load. Interestingly, smoking history did not independently influence CEA levels in our multivariable model, suggesting that tumor-driven biological factors dominate host-related factors in advanced disease settings.

Survival analyses demonstrated a consistent trend toward improved survival in patients with lower baseline CEA levels across molecular subtypes, although statistical significance was limited and appeared most evident in the rare alteration group. In patients with EGFR mutations, a numerical survival difference was observed, but it did not reach statistical significance. This finding may be explained by the substantial survival benefit associated with highly effective targeted therapies in EGFR-driven disease, which could attenuate the prognostic impact of baseline serum biomarker levels. In addition, subgroup sample size and event distribution may have limited statistical power to detect modest differences in survival. These findings suggest that the prognostic relevance of baseline CEA may be more informative in molecular subtypes with less effective targeted treatment options. Previous studies evaluating CEA in EGFR-mutant populations have similarly shown that baseline CEA may interact with treatment response, with some evidence suggesting a predictive value for EGFR-TKI efficacy [16,17], although the direction and clinical utility of this relationship remain to be clarified.

Consistent with established clinical data, KRAS-mutant disease was associated with poorer survival than EGFR-mutant tumors, reflecting the historically aggressive nature of KRAS-driven NSCLC. Importantly, baseline CEA retained independent prognostic significance after adjustment for driver mutation status, indicating that CEA provides complementary prognostic information beyond molecular profiling alone.

Several strengths of this study should be highlighted. This study represents one of the few multicenter analyses evaluating CEA levels across multiple oncogenic driver subtypes in metastatic NSCLC. The use of log-transformed CEA minimized bias related to extreme outliers and allowed for robust modeling of this highly skewed biomarker. In addition, the multicenter design enhances the real-world generalizability and reduces center-specific bias. Nevertheless, the limitations of this study should be acknowledged. The relatively high proportion of EGFR-mutant patients reflects the inclusion of a molecularly selected cohort with identified oncogenic alterations. This approach is aligned with the study objective of evaluating the association between baseline CEA levels and molecular subtypes. However, as the study population does not represent an unselected NSCLC cohort, the distribution of molecular alterations and related findings may not be fully generalizable to the broader NSCLC population. First-line treatment is strongly associated with driver mutation status in metastatic NSCLC, with EGFR-mutant patients predominantly receiving targeted therapies and KRAS-mutant patients mainly treated with chemotherapy. Therefore, the observed survival differences between molecular subtypes may partly reflect differences in treatment efficacy rather than intrinsic tumor biology alone. Given this strong association (collinearity) between treatment allocation and driver mutation status, the independent effect of molecular subtype on survival cannot be fully disentangled. This should be considered when interpreting the results. The retrospective design of this study introduces potential selection and information biases. Molecular testing methodologies varied between centers and over time, reflecting real-world practices but potentially contributing to heterogeneity. Differences in institutional testing standards and temporal changes in diagnostic approaches, including the transition from single-gene assays to more comprehensive NGS-based platforms, may have influenced mutation detection rates and the composition of molecular subgroups. Consequently, certain less common genomic alterations may have been underdetected in earlier years, potentially introducing ascertainment bias. This temporal and inter-center heterogeneity in molecular testing should be considered when interpreting the results. In addition, the relatively small sample size in certain molecular subgroups, particularly the rare or “other alterations” group, may have limited the statistical power and reduced the robustness of subgroup-specific analyses. Therefore, the associations observed between CEA levels and clinical outcomes in these smaller subgroups should be interpreted with caution. Moreover, PD-L1 status was unavailable for a substantial proportion of patients, representing an additional limitation of this retrospective study. PD-L1 expression may have influenced treatment decisions, particularly in KRAS-mutant disease where immunotherapy plays a central role. Therefore, the absence of PD-L1 data may represent a potential source of residual confounding in the survival analyses. ECOG performance status, a well-established prognostic factor in metastatic NSCLC, was not available in this study and was therefore not included in the multivariable model, which may represent a source of residual confounding. In addition, only patients with available baseline CEA measurements were included, regardless of molecular subgroup. While this approach ensured consistency across driver-defined groups, it may have introduced selection bias at the cohort level, as such patients may represent a subgroup with more comprehensive clinical evaluation and access to care, potentially influencing both survival outcomes and CEA distributions. While our overall findings support a potential prognostic role of baseline CEA in metastatic NSCLC, these results may not be fully generalizable across all molecular subtypes. Larger, prospective studies are warranted to validate these findings and to better define the prognostic value of CEA in less common molecular subgroups. Finally, only baseline CEA was evaluated, whereas dynamic changes in CEA during treatment, which have been shown to correlate with treatment response and disease progression, were not assessed [18,19].

## 5. Conclusions

In conclusion, baseline serum CEA levels differ across oncogenic driver alterations and are independently associated with the survival of patients with metastatic NSCLC. These findings suggest that CEA may reflect underlying tumor biology across molecular subtypes and may provide adjunctive clinical information. However, given the overlap between subgroups and the modest effect sizes, these results should be interpreted with caution. Prospective studies incorporating dynamic CEA monitoring and integrated molecular biomarkers are required.

## Figures and Tables

**Table 1:** Baseline patient, tumor, and treatment characteristics by driver alteration subgroup (EGFR, ALK, ROS1, KRAS, and other alterations) in the overall cohort (*n* = 332).

Variable	All patients (*n* = 332)	EGFR mutant (*n* = 180)	ALK positive (*n* = 45)	ROS1 positive (*n* = 27)	KRAS mutant (*n* = 65)	Other alterations * (*n* = 15)	*p* value
Sex							
Male	179 (53.9%)	68 (37.8%)	27 (60.0%)	19 (70.4%)	56 (86.2%)	9 (60.0%)	<0.001
Female	153 (46.1%)	112 (62.2%)	18 (40.0%)	8 (29.6%)	9 (13.8%)	6 (40.0%)	
Age at diagnosis (years)							
Median (min–max)	64 (33–89)	65 (35–89)	55 (33–80)	62 (33–80)	63 (40–87)	68 (46–84)	<0.001
Comorbidities							
Any comorbidity	163 (49.1%)	75 (41.7%)	25 (55.6%)	13 (48.1%)	40 (61.5%)	10 (66.7%)	0.031
Hypertension	109 (32.8%)	52 (28.9%)	13 (28.9%)	8 (29.6%)	26 (40.0%)	10 (66.7%)	0.036
Diabetes mellitus	55 (16.6%)	35 (19.4%)	3 (6.7%)	3 (11.1%)	10 (15.4%)	4 (26.7%)	0.179
Coronary artery disease	33 (10.0%)	17 (9.4%)	2 (4.5%)	4 (14.8%)	9 (13.8%)	1 (6.7%)	0.463
Asthma/COPD	61 (18.4%)	21 (11.7%)	9 (20.0%)	5 (18.5%)	21 (32.3%)	5 (33.3%)	0.003
Smoking/alcohol							
Smoking (active/ex)	167 (50.3%)	48 (26.7%)	29 (64.4%)	21 (77.8%)	59 (90.8%)	10 (66.7%)	<0.001
Pack-years, median (min–max)	35 (3–120)	30 (3–120)	36 (8–80)	35 (10–100)	40 (15–100)	37.5 (25.0–50.0)	0.150
Alcohol use	13 (3.9%)	3 (1.7%)	3 (6.7%)	1 (3.7%)	5 (7.7%)	1 (6.7%)	0.079
Tumor histology							
Adenocarcinoma	296 (89.2%)	171 (95.0%)	42 (93.3%)	25 (92.6%)	49 (75.4%)	9 (60.0%)	<0.001
Squamous cell carcinoma	31 (9.3%)	8 (4.4%)	2 (4.4%)	1 (3.7%)	14 (21.5%)	6 (40.0%)	
Other **	5 (1.5%)	1 (0.6%)	1 (2.2%)	1 (3.7%)	2 (3.1%)	0 (0.0%)	
Stage at diagnosis							
I	17 (5.1%)	15 (8.3%)	1 (2.2%)	1 (3.7%)	0 (0.0%)	0 (0.0%)	0.046
II	15 (4.5%)	10 (5.6%)	4 (8.9%)	1 (3.7%)	0 (0.0%)	0 (0.0%)	
III	38 (11.4%)	23 (12.8%)	2 (4.4%)	5 (18.5%)	7 (10.8%)	1 (6.7%)	
IV	262 (78.9%)	132 (73.3%)	38 (84.4%)	20 (74.1%)	58 (89.2%)	14 (93.3%)	
Metastatic sites count							
Median (min–max)	2 (0–6)	2 (0–6)	2 (0–5)	2 (1–4)	2 (0–5)	3 (1–5)	0.255
Metastatic pattern							
Liver metastasis	71 (21.4%)	34 (18.9%)	13 (28.9%)	6 (22.2%)	14 (21.5%)	4 (26.7%)	0.656
Lung metastasis	167 (50.3%)	89 (49.4%)	23 (51.1%)	11 (40.7%)	33 (50.8%)	11 (73.3%)	0.374
Brain metastasis	94 (28.3%)	51 (28.3%)	15 (33.3%)	5 (18.5%)	20 (30.8%)	3 (20.0%)	0.638
Bone metastasis	168 (50.6%)	94 (52.2%)	20 (44.4%)	16 (59.3%)	31 (47.7%)	7 (46.7%)	0.737
Adrenal metastasis	72 (21.7%)	32 (17.8%)	9 (20.0%)	5 (18.5%)	19 (29.2%)	7 (46.7%)	0.062
Distant LN metastasis	56 (16.9%)	24 (13.3%)	13 (28.9%)	6 (22.2%)	12 (18.5%)	1 (6.7%)	0.104
Pleural metastasis	65 (19.6%)	40 (22.2%)	12 (26.7%)	6 (22.2%)	3 (4.6%)	4 (26.7%)	0.005
Local lung recurrence	49 (14.8%)	38 (21.1%)	5 (11.1%)	4 (14.8%)	1 (1.5%)	1 (6.7%)	0.001
PD-L1-TPS							
Unknown	163 (49.1%)	87 (48.3%)	25 (55.6%)	12 (44.4%)	32 (49.2%)	7 (46.7%)	0.297
<1%	88 (26.5%)	50 (27.8%)	11 (24.4%)	7 (25.9%)	13 (20.0%)	7 (46.7%)	
1–49%	61 (18.4%)	37 (20.6%)	6 (13.3%)	5 (18.5%)	12 (18.5%)	1 (6.7%)	
≥50%	20 (6.0%)	6 (3.3%)	3 (6.7%)	3 (11.1%)	8 (12.3%)	0 (0.0%)	
First-line treatment group							
No systemic therapy	4 (1.2%)	0 (0.0%)	0 (0.0%)	0 (0.0%)	4 (6.2%)	0 (0.0%)	<0.001
Chemotherapy	125 (37.7%)	33 (18.3%)	11 (24.4%)	13 (48.1%)	56 (86.2%)	12 (80.0%)	
Chemotherapy + IO	2 (0.6%)	0 (0.0%)	0 (0.0%)	0 (0.0%)	2 (3.1%)	0 (0.0%)	
IO monotherapy	3 (0.9%)	0 (0.0%)	0 (0.0%)	0 (0.0%)	3 (4.6%)	0 (0.0%)	
TKI	198 (59.6%)	147 (81.7%)	34 (75.6%)	14 (51.9%)	0 (0.0%)	3 (20.0%)	
Metastatic treatment lines							
Median (min–max)	2 (0–8)	2 (0–8)	1 (1–7)	2 (1–4)	2 (0–8)	2 (1–5)	0.333
CEA							
Median (min–max)	6.98 (0.20–5505.54)	10.365 (0.650–3282.050)	4.00 (0.20–5505.54)	6.00 (1.00–228.80)	5.80 (0.30–587.70)	3.00 (1.02–55.00)	0.001

* Other mutations include BRAF V600 (*n* = 3) and non-V600 (*n* = 1), HER2 overexpression (*n* = 2), TP53 mutation (*n* = 7), PTEN mutation (*n* = 1), PIK3CA mutation (*n* = 1). ** Other histologies include adenosquamous carcinoma (*n* = 2) and NSCLC—Not Otherwise Specified (*n* = 3).

**Table 2:** Multivariable generalized linear model for log-transformed CEA (lnCEA).

Predictor	Comparison (Reference)	GMR (exp [B])	95% CI (GMR)	*p*-value
Driver alteration	ALK vs. EGFR	0.500	0.282–0.887	0.018
	KRAS vs. EGFR	0.588	0.289–1.199	0.144
	ROS1 vs. EGFR	0.555	0.317–0.971	0.039
	Other vs. EGFR	0.363	0.143–0.919	0.033
Smoking status	Active/ex-smoker vs. non-smoker	0.854	0.556–1.311	0.471
Histology	Squamous cell carcinoma vs. adenocarcinoma	0.432	0.224–0.833	0.012
	Other histology vs. adenocarcinoma	2.105	0.477–9.291	0.326
Number of metastatic sites	Per +1 metastatic site	1.206	1.031–1.410	0.019

**Table 3:** Univariate Cox regression for mortality.

Variable	HR	95% CI	*p*-value
ln(CEA) (per 1-unit)	1.076	1.001–1.157	0.046
Driver: ALK vs. EGFR	0.887	0.566–1.392	0.603
Driver: ROS vs. EGFR	1.203	0.720–2.008	0.481
Driver: KRAS vs. EGFR	2.689	1.933–3.741	<0.001
Driver: other vs. EGFR	1.700	0.934–3.095	0.082
Metastatic site count (per +1)	1.275	1.141–1.424	<0.001
Histology: SCC vs. Adeno	1.505	0.988–2.294	0.057
Histology: Other vs. Adeno	2.353	0.872–6.351	0.091
Age at diagnosis (per year)	1.014	1.002–1.026	0.026
Sex: Female vs. Male	0.568	0.432–0.748	<0.001
Smoking: active/ex vs. never	1.613	1.232–2.113	<0.001
Brain metastasis: yes vs. no	1.272	0.945–1.714	0.113
Liver metastasis: yes vs. no	1.297	0.949–1.772	0.103
Bone metastasis: yes vs. no	1.476	1.127–1.934	0.005
Treatment: Chemo vs. none	0.425	0.134–1.348	0.146
Treatment: Chemo + IO vs. none	0.549	0.057–5.301	0.604
Treatment: IO mono vs. none	0.525	0.087–3.157	0.482
Treatment: TKI vs. none	0.242	0.076–0.767	0.016

**Table 4:** Multivariate Cox regression for mortality.

Variable	HR (95% CI)	*p*-Value
lnCEA (per 1-unit increase)	1.149 (1.059–1.246)	<0.001
Age at diagnosis (per year)	1.012 (0.998–1.025)	0.086
Sex: Female vs. Male	0.623 (0.452–0.860)	0.004
Metastatic site count (per +1)	1.273 (1.137–1.426)	<0.001
Smoking: active/ex vs. never	1.164 (0.807–1.679)	0.417
Histology: SCC vs. Adenocarcinoma	1.255 (0.789–1.994)	0.264
Histology: Other ^†^ vs. Adenocarcinoma	2.008 (0.730–5.525)	0.177
Driver: ALK vs. EGFR	0.830 (0.504–1.369)	0.466
Driver: ROS1 vs. EGFR	1.095 (0.622–1.928)	0.754
Driver: KRAS vs. EGFR	2.301 (1.509–3.510)	<0.001
Driver: Other vs. EGFR	1.391 (0.714–2.709)	0.332

^†^ Other histologies include adenosquamous carcinoma (*n* = 2) and NSCLC- Not Otherwise Specified (*n* = 3).

## Data Availability

The datasets generated and/or analyzed during the current study are not publicly available due to patient privacy and ethical restrictions but are available from the corresponding author on reasonable request.

## Abbreviations

The following abbreviations are used in this manuscript:

NSCLC	Non-Small Cell Lung Cancer
CEA	Carcinoembryonic Antigen
OS	Overall Survival
EGFR	Epidermal Growth Factor Receptor
ALK	Anaplastic Lymphoma Kinase
ROS1	ROS Proto-Oncogene 1
KRAS	Kirsten Rat Sarcoma Viral Oncogene Homolog
BRAF	B-Raf Proto-Oncogene
MET	Mesenchymal-Epithelial Transition
PD-L1	Programmed Death-Ligand 1
TPS	Tumor Proportion Score
IHC	Immunohistochemistry
PCR	Polymerase Chain Reaction
FISH	Fluorescence In Situ Hybridization
NGS	Next-Generation Sequencing
HR	Hazard Ratio
CI	Confidence Interval
GLM	Generalized Linear Model
